# Validity of the ACTS intimate partner violence screen in antenatal care: a cross sectional study

**DOI:** 10.1186/s12889-021-11781-x

**Published:** 2021-09-24

**Authors:** K. Hegarty, J. Spangaro, M. Kyei-Onanjiri, J. Valpied, J. Walsh, J. Chapman, J. Koziol-McLain

**Affiliations:** 1 Department of General Practice, Faculty of Medicine, Dentistry and Health Sciences, The University of Melbourne (and The Royal Women’s Hospital), 780 Elizabeth St, Carlton, VIC 3053 Australia; 2 School of Health and Society Wollongong, University of Wollongong, New South Wales Wollongong, Australia; 3 The Royal Women’s Hospital, Centre for Family Violence Prevention, 20 Flemington Rd, Parkville, Victoria Australia; 4 School of Clinical Sciences Auckland, Auckland University of Technology, Auckland, New Zealand

**Keywords:** Intimate partner violence, Domestic violence, Routine screening, Screening tool, Antenatal care, Pregnancy

## Abstract

**Background:**

Intimate partner violence (IPV) is a major public health problem with harmful consequences. In Australia, there is no national standard screening tool and screening practice is variable across states. The objectives of this study were to assess in the antenatal healthcare setting: i) the validity of a new IPV brief screening tool and ii) women’s preference for screening response format, screening frequency and comfort level.

**Methods:**

One thousand sixty-seven antenatal patients in a major metropolitan Victorian hospital in Australia completed a paper-based, self-administered survey. The survey included four screening items about whether they were Afraid/Controlled/Threatened/Slapped or physically hurt (ACTS) by a partner or ex-partner in the last 12 months; and the Composite Abuse Scale (reference standard). The ACTS screen was presented firstly with a binary yes/no response format and then with a five-point ordinal frequency format from ‘never’ (0) to ‘very frequently’ (4). The main outcome measures were test statistics of the four-item ACTS screening tool (sensitivity, specificity, predictive values, and area under the curve) against the reference standard and women’s screening preferences.

**Results:**

Twelve-month IPV prevalence varied depending on the ACTS response format with 8% (83) positive on ACTS yes/no format, 12.8% (133) positive on ACTS ordinal frequency format and 10.5% (108) on the reference Composite Abuse Scale. Overall, the ACTS screening tool demonstrated clinical utility for the ordinal frequency format (AUC, 0.80; 95% CI = 0.76 to 0.85) and the binary yes/no format (AUC, 0.74, 95% CI = 0.69 to 0.79). The frequency scale (66%) had greater sensitivity than the yes/no scale (51%). The positive and negative predictive values were 56 and 96% for the frequency scale and 68 and 95% for the yes/no scale. Specificity was high regardless of screening question response options. Half (53%) of the women categorised as abused preferred the yes/no scale. Around half of the women (48%, 472) thought health care providers should ask pregnant women about IPV at every visit.

**Conclusions:**

The four-item ACTS tool (using the frequency scale and a cut-off of one on any item) is recommended for written self-administered screening of women to identify those experiencing IPV to enable first-line response and follow-up.

**Supplementary Information:**

The online version contains supplementary material available at 10.1186/s12889-021-11781-x.

## Background

Intimate partner violence (IPV) is a major public health problem with harmful consequences on the health of women [1], and their unborn babies and children [2, 3]. Globally, it is estimated that about 1 in 3 women have experienced physical and/or sexual violence by an intimate partner [4]. IPV is common during pregnancy, with estimates varying from 3 to 29% depending on the measure used, and for many women, violence begins or escalates during pregnancy and the postpartum period [5–7]. Women experiencing IPV have higher risk for adverse maternal and perinatal health outcomes including postnatal depression, perceived stress [8]; unintended pregnancies, abortions [9]; low birth weight and preterm births [10].

Given the increased contact with healthcare providers during pregnancy, antenatal care presents a unique opportunity to enquire routinely about IPV [11]. A Cochrane systematic review [12] suggests that IPV screening and initial response by a health professional increases identification with no increase in referrals or changes in women’s experience of violence or wellbeing. However, in antenatal care there may be sufficient evidence to recommend screening all women attending, with two antenatal studies [13, 14] showing improvement in some outcomes for women [12]. Despite increased efforts to reduce IPV and its negative health consequences, it is not consistently screened for in antenatal care across the world [15, 16]. Although there are many barriers to effective identification and response for women experiencing IPV [16], one factor increasing a health professional’s likelihood of screening for IPV is having a set of scripted questions [17–20]. The use of a validated tool suitable to antenatal settings may facilitate consistent screening but also allow comparisons across health facilities and changes over time for quality improvement purposes [17].

In evaluating IPV screening tools, the balancing act between correctly identifying those experiencing IPV (the true positive rate or sensitivity) and eliminating those not experiencing it (true negative rate specificity) is difficult when dealing with a social problem rather than a biomedical disease with a straight-forward diagnostic gold standard [21]. Thus, there are several analysis issues including that there is no agreed upon gold standard for IPV measurement and pre-test prevalence will alter the positive predictive value of the screening tool. There are also interpretation issues, such as the reductionist approach of IPV screening tools in which women are dichotomised into abused or non-abused categories. Among any group of women who do not report IPV on a particular tool, will be some who have experienced abusive behaviours but do not wish to label themselves as abused. This should be respected and understood.

With IPV it is important to maximise reach to those who have been abused by a partner so that support can be offered, that is, there is a need to maximise the true positive rate. However, there are implications of both false positives (overidentifying cases of IPV) and false-negatives (missing cases of IPV). An ‘over-inclusive’ IPV screen, will mean that some women will be identified as experiencing abuse when the behaviours they experience are not consistent with the current understanding of the coercive controlling dynamics of IPV. For these women (returning a false positive IPV screen), there is a risk of being labelled as someone who is experiencing IPV resulting in unnecessary use of intervention resources. Where the prevalence of the condition of interest is very low, as it often is with screening for IPV in the last 12 months, a test has to be highly specific to reduce the number of false-positive results to an acceptable level [22].

Gaps remain on the most effective ways of screening to identify those affected by IPV and what screening tools to use [23]. A 2009 systematic review showed that the psychometric properties varied across IPV screening tools and settings [24]. This review reported that the most studied screening tools were the Hurt, Insult, Threaten, and Scream (HITS, sensitivity 30–100%, specificity 86–99%); the Woman Abuse Screening Tool (WAST, sensitivity 47%, specificity 96%); the Partner Violence Screen (PVS, sensitivity 35–71%, specificity 80–94%); and the Abuse Assessment Screen (AAS, sensitivity 93–94%, specificity 55–99%). Internal reliability (HITS, WAST); test–retest reliability (AAS); concurrent validity (HITS, WAST); discriminant validity (WAST); and predictive validity (PVS) were also assessed, however the authors concluded that no single IPV screening tool had well-established psychometric properties. A 2016 systematic review [23] found 10 IPV screening tools and recommended three as having stronger psychometric values, assessing physical, emotional and sexual IPV and having been validated against a reference standard: Women Abuse Screen Tool (WAST), Humiliation, Afraid, Rape and Kick (HARK) and Abuse Assessment Screen (AAS). A strength of the tools is inclusion of questions about fear, which has the potential to identify the majority of women experiencing serious IPV [25].

However, there are several issues with these tools including length, inclusion of sexual violence behaviours, scoring resulting in varied prevalence among pregnant women and lack of addressing coercive control behaviours. The eight item tool WAST [26], though comprehensive, is longer than most tools and this is a consideration in light of findings about the importance of tool brevity e.g., HARK four-item tool [20, 27]. Both tools also include items about sexual violence, which is common in abusive relationships, yet in the context of an initial screen, may be difficult for health providers to ask about and a particularly challenging form of abuse for women to name [28, 29]. The five item AAS has a simple scoring system and has been validated in perinatal settings [30–36] but has demonstrated a large range of prevalence from 2.8% [34] to 35.5% [35] for IPV during the antenatal period and up to 41% [31] for any history of IPV among a sample of pregnant women. Further, none of these scales (i.e., WAST, HARK, AAS) capture coercive control which is seen as an important part of the pattern of IPV [37].

Systematic reviews have shown that women find screening tools acceptable [38, 39]; however, an additional characteristic of IPV screening tools that is understudied is the format of item responses. Women are generally asked to report the occurrence of abusive behaviours in the past 12 months in either a binary response format (yes or no) or an ordinal frequency format. While HARK [27], AAS [40], and PVS [41] response choices are yes or no, the WAST has three options (‘often’, ‘sometimes’ and ‘never’) [42] and HITS has five options (‘never’, ‘rarely’, ‘sometimes’, ‘fairly often’, ‘frequently) [43]. It is not known whether response format affects IPV screening tool validity. It is also not known whether women prefer to respond to screening questions with a binary yes/no or have a range of frequency options. IPV screen length, response options and scoring may all impact on both validity and ease of use for health practitioners and women clients.

Recognising the shortcomings of current IPV screening tools for use in antenatal care, we developed the brief ACTS tool through reviewing items on existing tools and a consensus discussion amongst the authors [17]. In this paper, we introduce the ACTS tool and present our findings of initial tool testing. Our aim was to test in antenatal care i) the accuracy of the new IPV screening tool and ii) how women prefer to be asked about IPV. We present test statistics (sensitivity, specificity, negative and positive predictive values and area under the receiver operating curve [AUC]) against the reference standard Composite Abuse Scale (CAS) [44] and the utility of the ACTS tool with two alternative response formats. We also assess women’s preference for IPV screen response format and frequency of asking, along with their comfort level in being screened.

## Methods

This analysis was part of the larger study - *Sustainability of identification and response to domestic violence in antenatal care* (the SUSTAIN study), funded by the Australian Research Organisation for Women’s Safety. Detailed methods for the SUSTAIN study are reported elsewhere [17]. Briefly, participants were antenatal patients in a major metropolitan Victorian hospital who completed a paper-based, self-administered survey while they waited for their antenatal clinic appointments.

### Data collection and recruitment

Patients were approached in the antenatal waiting room between May and July 2018 and asked if they were accompanied by a partner, family member or other person(s). Those who responded ‘yes’ were thanked and not invited to participate to minimize potential risks to the safety of women from possible perpetrator awareness. Women presenting unaccompanied who were at least 16 years of age and were proficient in written English, Arabic, Mandarin or Cantonese (the four most common languages spoken at the hospital) were offered information about the SUSTAIN study.

The SUSTAIN survey included six sections: pregnancy and pregnancy care; health and well-being; relationships and safety; supports; personal and household details; and views about the survey [17]. Within the relationships and safety section were the IPV screening four items relating to partner or ex-partner behaviours: Afraid, Controlled, Threatened to physically hurt, Hit, Slapped, or physically hurt (ACTS) (see Table 2). These four items were presented twice consecutively within the survey, with two response formats. We advised women that there would be repetition of questions and encouraged them to answer all formats. The first format presented a binary ‘yes’ (coded 1) or ‘no’ (coded 0) response to each of the four items. The second format presented a five-point ordered response based on frequency of the behaviours (never = 0, rarely = 1, sometimes = 2, frequently = 3, very frequently = 4). An additional question asked which of these response formats women preferred when being asked about IPV (Thinking about question 2a and 2b, which way would you prefer to be asked? 2a [answering with yes or no], 2b [answering how often]). Another question asked how often they would prefer to be asked ACTS during pregnancy (When do you think is the best time during pregnancy for health care providers to ask about domestic violence? [at the first visit only; at some visits i.e., first, 28 weeks and 36 weeks; at every visit; I don’t think they should ask]). The SUSTAIN survey then included the Composite Abuse Scale (CAS) [44] as an IPV reference standard. The CAS is a comprehensive, multidimensional measure of IPV covering physical, sexual, and psychological abuse. Women self-report their experience of 30 abuse acts in the past 12 months on a six-point frequency scale from ‘never’ (0) to ‘daily’ (5). It has four dimensions: Severe Combined Abuse, Emotional Abuse, Physical Abuse, and Harassment that provide four categories of abuse for an individual woman’s experience of IPV [44].

### .Analysis

A ‘yes’ response to one or more of the four binary format ACTS questions was considered a positive screen for IPV. A response of ‘rarely’ or higher for one or more of the four frequency format ACTS questions was considered a positive screen for IPV. For the reference standard CAS, scoring on one or more of the four abuse categories was considered positive for experiencing IPV [17]. A small number of participants were unable to be classified due to missing screening and/or CAS items. Sensitivity, specificity, and predictive values of the ACTS screening tools were assessed against the CAS. Receiver operating characteristic (ROC) curve analysis was conducted to assess the overall accuracy of the ACTS screening questions using CAS as the reference. For each ROC curve, the AUC is reported when plotting sensitivity against 1-specificity. AUC of greater than 0.75 is generally considered clinically useful [45].

Participants were assured participation was voluntary and choosing not to participate would not impact their care. A distress protocol and information resources were available for both women and researchers involved in the study. Where women screened positive for IPV, they were prompted to speak to their midwife or social worker, or IPV service.

## Results

The survey was completed by 1067 pregnancy care patients aged between 18 and 48 years, representing a response rate of 76.2%. Approximately half (49%, 515) of participants were pregnant with their first child and nearly all (97%; 1008) had a current partner. The study sample was linguistically diverse with about 27% (273) of participants having a first language other than English and 1% [10] identified as Aboriginal or Torres Strait Islander women. Over 90% (931) had completed at least Year 12 education (Table 1).

**Table 1:** Demographic characteristics of participants (*N* = 1067)

	Mean	SD
Age in years (*n* = 942, min =18, max = 48)	33.2	4.5
Weeks pregnant (*n* = 991, min = 6, max = 41)	27.0	7.6
	**N**	**%**
First baby	515	49.2
Has current partner	1008	96.6
Married	707	69.8
Defacto (living with partner)	263	26.0
Type of care received		
Hospital care	462	54.7
Shared care	204	24.2
Midwifery care	154	18.3
Medical care	43	5.0
Specialist clinic	63	7.0
Community clinic	2	0.2
Attending first appointment	218	21.0
Aboriginal or Torres Strait Islander	10	1.0
Born outside Australia	455	45.0
English not first language	273	27.1
Finished school to Year 12	931	92.4
Completed at least a university degree	729	72.2
Has a Health Care Card	289	28.6
Ease of managing on current income		
Easily	411	40.3
Not too bad	404	39.7
Difficult some of the time	175	17.2
Difficult all the time	26	2.6
Impossible	3	0.3

Denominators vary due to missing data

### Rates of IPV and abusive behaviours experienced by participants

The proportion of women screening positive for IPV was 8% (*n* = 83) based on the four-item binary (yes/no) response format ACTS and 12.8% (*n* = 133) for the ordinal frequency response format (Table 2). These rates compare to 10.5% (*n* = 108) of women categorised as positive for IPV experience using the CAS reference standard. While both ACTS IPV screen prevalence estimates approximated the referent standard (overlapping confidence intervals), the estimated prevalence of the dichotomous response format tool was lower than for the frequency response format.

**Table 2:** Abusive behaviours experienced by participants in past 12 months

	IPV Positive		
	N	%	95% CI
**ACTS Screening Tool – Binary Yes/No format**^**a**^**(*n*** **= 1042)**			
*Has partner or ex-partner…*			
Done something that made you feel afraid?	55	5.3	
Controlled your day-to-day activities or put you down?	44	4.2	
Threatened to hurt you in any way?	17	1.6	
Hit, slapped, kicked, or otherwise physically hurt you?	13	1.2	
**Any of the above**^**b**^	83	8.0	6.3, 9.6
**ACTS Screening Tool – Ordinal Frequency format**^**c**^**(n = 1042)**			
*Has partner or ex-partner…*			
Done something that made you feel afraid?	94	9.0	
Controlled your day-to-day activities or put you down?	81	7.8	
Threatened to hurt you in any way?	26	2.5	
Hit, slapped, kicked, or otherwise physically hurt you?	31	3.0	
**Any of the above**^**d**^	133	12.8	10.7, 14.8
**Composite Abuse Scale (*n*** **= 1029)**			
*Scored positive for the following category of abuse…*			
Severe Combined Abuse	28	2.7	
Emotional Abuse /Harassment alone	57	5.5	
Physical Abuse alone	5	0.5	
Physical and Emotional Abuse/Harassment	18	1.7	
**Any category of abuse on CAS**	108	10.5	8.6, 12.4

Denominators vary due to missing data. *ACTS* Afraid/Controlled/Threatened/Slapped or otherwise physically hurt, *CI* confidence intervals

^a^‘*Yes*’ in response to question, “In the last year, has a partner or ex-partner…”, where response options for each item were *Yes* (scored 1) or *No* (scored 0)

^b^Score of 1 (*Yes*) on one or more of the ACTS Screening Tool items

^c^ ‘*Rarely*’ or above in response to question, “In the last year, how often has a partner or ex-partner…”, where response options for each item were on a five-point Likert scale (*Never* = 0, *Rarely* = 1, *Sometimes* = 2, *Frequently* = 3, *Very frequently* = 4)

^d^Score ≥ 1 (*Rarely*) on one or more of the ACTS Screening Tool items

### Accuracy of ACTS IPV screen

The test performance of the four individual ACTS screen items and the overall screen, judged against the referent CAS, by response format, followed a consistent pattern (see Table 3). The sensitivity was higher with the ordinal frequency response format while the specificity was higher for the binary response format. Given the more stringent binary response (resulting in fewer women categorised as abused) compared to the ordinal frequency format, it is not surprising that the positive predictive value was higher for the binary format (0.68) compared to the ordinal (0.56). This is due to the yes/no response format being associated with fewer false positives. Each of the ACTS’ four items and overall, regardless of response format, performed well in identifying women who did not experience IPV based on the CAS, with negative predictive values of .95 (binary response) and .96 (ordinal frequency response).

**Table 3:** ACTS IPV screening test characteristics in predicting cases positive for any category of abuse on the Composite Abuse Scale (total *N* = 1011, number of participants meeting reference criteria = 104^a^)

Predictor (screening item)	Response type^**b**^	PPV	NPV	Sensitivity	Specificity	Area under curve				
						Area	95% CI^**c**^		***Chi***^***2 d***^	***p***
**Individual items:**										
Done something that made you feel afraid?	*Yes/No*	0.65	0.93	0.32	0.98	0.65	(0.60 to	0.69)		
	*Frequency*	0.56	0.94	0.47	0.96	0.71	(0.67 to	0.76)	10.25	.0014
Controlled your day to day activities … or put you down?	*Yes/No*	0.79	0.93	0.32	0.99	0.65	(0.61 to	0.70)		
	*Frequency*	0.63	0.94	0.46	0.97	0.72	(0.67 to	0.76)	12.45	.0004
Threatened to hurt you in any way?	*Yes/No*	0.81	0.91	0.13	> 0.99	0.56	(0.53 to	0.59)		
	*Frequency*	0.83	0.92	0.23	0.99	0.61	(0.57 to	0.65)	9.69	.0019
Hit, slapped, kicked or otherwise physically hurt you?	*Yes/No*	0.92	0.91	0.12	> 0.99	0.56	(0.53 to	0.59)		
	*Frequency*	0.81	0.92	0.20	0.99	0.60	(0.56 to	0.64)	8.73	.0031
**Overall ACTS screen:**										
Positive screen (one or more positive items)^e^	*Yes/No*	**0.68**	**0.95**	**0.51**	**0.97**	**0.74**	**(0.69 to**	**0.79)**		
	*Frequency*	**0.56**	**0.96**	**0.66**	**0.94**	**0.80**	**(0.76 to**	**0.85)**	**11.18**	**.0008**

*CI* confidence interval, *PPV* positive predictive value, i.e. proportion of positively screened participants who met criteria for any abuse category on Composite Abuse Scale, *NPV* negative predictive value, i.e. proportion of negatively screened participants who did not meet criteria for any abuse category on Composite Abuse Scale; Sensitivity = proportion of abused participants who screened positive on the predictor; Specificity = proportion of non-abused participants who screened negative on the predictor. ^a^Missing cases were excluded listwise. ^b^Items were presented to all participants in two formats: *Yes/No format -* Participants were asked, “In the last year, has a partner or ex-partner…”, and response options for each item were yes (scored 1) or no (scored 0); *Frequency format -* Participants were asked, “In the last year, how often has a partner or ex-partner…”, and responses for each item were on a five point scale (Never = 0, Rarely = 1, Sometimes = 2, Frequently = 3, Very frequently = 4); in both formats a participant screened positive if they had a score of at least 1. ^c^Asymptotic 95% confidence interval. ^d^Chi2 for difference between area under curve when using binary versus frequency response format. ^e^Screened positive (score of at least 1) on any of the four screening items. ^f^Screened positive (score of at least 1) on “Done something that made you feel afraid?” or “Controlled your day-to-day activities (e.g. who you see, where you go) or put you down?”

The ACTS screening tool demonstrated good overall accuracy demonstrated by an area under the ROC curve of 0.80 (95% CI = 0.76 to 0.85) for the ordinal frequency response and 0.74, (95% CI = 0.69 to 0.79) for the binary response (see Table 3 and Additional file Figure 1).

### Women’s preferences for IPV screening response format, frequency of enquiry and comfort to be asked

A small majority (60%) of participants indicated a preference for the ACTS screening tool option with the binary yes/no response format over the ordinal frequency format (Table 4). Comparing those women exposed to IPV or not, 53% of women who have experienced IPV (53, 95% CI = .43, .62) preferred the binary yes/no response format compared to 61% (489, 95% CI = 0.58, 0.62) of women not experiencing IPV.

**Table 4:** Participant preferences regarding IPV screening

	IPV Positive (CAS)		IPV Negative (CAS)		TOTAL	
	n	%	n	%	n	%
**IPV screen response format preference**	*n* = 100		*n* = 802		*n* = 902	
Binary yes/no format^a^	53	53	491	61	544	60.3
**When should provider ask about IPV**	*n* = 104		*n* = 873		*n* = 977	
At first visit only	12	12	129	15	141	14
At some visits	37	36	301	34	338	35
At every visit	48	46	417	48	465	48
Don’t think they should ask	7	7	26	3	33	3
**Level of comfort talking about IPV**^**b**^	*n* = 54		*n* = 238		*n* = 292	
Very comfortable	7	13	78	33	85	29
Comfortable	19	35	80	34	99	34
Neutral	11	20	36	15	47	16
Uncomfortable	10	19	35	15	45	15
Very uncomfortable	7	13	9	4	16	5

^a^ Preferred binary yes/no response format rather than ordinal frequency response. ^b^ Only includes participants who responded to this item by selecting a comfort-level response option; An additional 722 participants instead selected the option, “I have not experienced this issue”

The majority of participants (82%) supported health care providers asking about IPV screening more than once (‘at some visits’ or ‘at every visit’) during antenatal care, compared to at only the first visit (14%), or not at all (3%) (Table 4). While most women were comfortable talking about IPV, one out of every three women (31%) exposed to IPV (CAS positive) indicated they were uncomfortable or very uncomfortable talking with health providers about experiencing fear of a current or ex-partner (Table 4).

## Discussion

This study assessed the validity of a new four-item ACTS IPV screening tool developed for use in antenatal healthcare settings and the findings are promising. In this sample of 1067 women, the ACTS screening tool with an ordinal frequency response format, provided a balance of sensitivity and specificity, correctly identifying 66% of women with IPV and 94% of women without IPV. The ACTS screening tool also demonstrated clinical utility, with 56% of women with a positive screen and 96% of women with a negative screen correctly classified based on the referent Composite Abuse Scale (predictive values). These predictive values are dependent on the pre-test prevalence, which in this sample was around 1 in 10 women attending the antenatal care setting. Similar trends were observed for the binary response format (51% sensitivity and 97% specificity with 68% of true IPV-positive cases and 95% of true IPV-negative cases). These figures are higher than with other tools including the WAST (sensitivity 47%, specificity 96%) and PVS (sensitivity 35–71%, specificity 80–94%) [24].

The ACTS screening tool was efficient at ruling out women who were not experiencing IPV but less accurate for detecting women experiencing IPV. Decisions regarding optimal use of the tool in healthcare settings should be informed by the general objectives of screening. In fact, the high NPV offers clinicians the confidence that women who are not experiencing IPV are more likely to be ruled out during screening than those experiencing IPV. This is a strength of the tool, as it enables clinicians to differentiate between these two groups of women and may allow them to direct more attention and resources through further assessment and follow-up to those who screened positive.

With respect to how screening questions should be framed or asked at antenatal visits, participants categorised as abused were split on which format of response they preferred, while more participants in the non-abused category preferred the binary format. This may be partly attributable to the fact that women in the non-abused category might find yes/ no questions less demanding. On the other hand, women who experience abuse may appreciate being able to disclose in steps, for example, replying “sometimes” rather than committing to the ‘all or nothing’ approach that yes or no requires. This is consistent with the conclusion of an analysis of women’s perspectives on IPV screening questions showing that answers to questions were rarely “yes” or “no” and thus midwives were often unclear whether women’s responses constituted IPV [46]. With regard to frequency of assessing for IPV, over three quarters of women indicated that screening questions should be asked more than once throughout the antenatal period (including 48% of respondents who preferred being asked at every visit). This is consistent with the knowledge that for IPV screening tools to be effective, they need to be repeated during the antenatal period and postnatally, with an ability to document clearly previous answers so women are not repeatedly asked for the same information [47]. Indeed, screening metrics aside, it is vital that screening tools do not dominate decision making but rather complement professional judgement of trained clinicians who are supported by their workplaces [48]. Addressing the sensitive topic of IPV requires trained clinicians knowledgeable about dynamics of IPV, structural entrapment, impacts on families and available specialist resources.

### Strengths and limitations

This study included more than 1,000 pregnant women, providing the opportunity for robust analysis of the ACTS tool. There are, however, study limitations to consider. While the sample was large and somewhat diverse, the majority of women in this urban setting were well-educated and financially secure. It is important that policy addressing the health response to IPV address the needs of those experiencing IPV along with a multitude of structural social and economic disadvantages. While we were able to consider women’s preferences for response formats by abuse status, there are likely subgroups of women who require bespoke IPV assessment and response with associated training tailored to women’s backgrounds and context. Research drawing on traditional ways of knowing would be needed to explore a safe and effective assessment and response for Aboriginal and Torres Strait Islander women [49]. In addition, for safety reasons, only women presenting to their antenatal care visit unaccompanied were eligible to participate, which may have excluded some groups of women. The IPV prevalence estimates, therefore, may not be representative of the general antenatal population. In addition, in the SUSTAIN survey, the binary response ACTS tool preceded the tool with ordinal frequency (static order rather than randomised), which was then followed by the Composite Abuse Scale. While women were informed that they would be presented questions in several different ways, the results may be influenced by a testing effect. Finally, further research would be warranted testing the ACTS tool across clinical settings with diverse samples and using different modes of delivery (verbal, written, electronic). Improving the sensitivity of the tool may require such research, including the use of qualitative methods to understand how women interpret the questions and response formats.

## Conclusions

We conclude that the ACTS tool provides a new clinically useful resource to identify women in the antenatal setting most likely to be experiencing IPV, who could potentially benefit from a health system response [11]. The introduction of this new brief IPV screening tool, that includes controlling behaviour, offers the opportunity for antenatal clinicians to address this important determinant of health [17]. However, evidence informed practice requires that clinicians are supported by a health system that offers private spaces, mandatory staff training, clear protocols and referral pathways, and leadership [1]. Policies need to encompass all aspects of this health system support.

## Supplementary Information

**Additional file 1: Figure 1.** Receiver Operating Characteristic curves for the ACTS screening tool (binary “Yes/No” and Likert-scale “Frequency” formats). Description of data: Receiver Operating Characteristic curves for the ACTS screening tool when presented as binary “Yes/No” format and Likert-scale “Frequency” format

## Data Availability

The data that support the findings of this study are available from the corresponding author, but restrictions apply to the availability of these data, which were used under license for the current study, and so are not publicly available. Data are however available from the corresponding author upon reasonable request.

## Abbreviations

AAS: Abuse Assessment Screen
ACTS: Afraid/Controlled/Threatened/ Slapped or physically hurt
AUC: Area under the curve
CAS: Composite Abuse Scale
HARK: Humiliation, Afraid, Rape and Kick
HITS: Hurt, Insult, Threaten, and Scream
IPV: Intimate partner violence
PVS: Partner Violence Screen
ROC: Receiver operating characteristic
SUSTAIN: Sustainability of identification and response to domestic violence in antenatal care
WAST: Woman Abuse Screening Tool
