# Supplementary material for: Validity of the ACTS intimate partner violence screen in antenatal care: a cross sectional study
Source: BMC Public Health. 2021 Sep 24;21:1733. doi: 10.1186/s12889-021-11781-x (PMC8461928; doi:10.1186/s12889-021-11781-x)

**Figure 1:**  **Receiver Operating Characteristic curves for the ACTS screening tool (binary “Yes/No” and Likert-scale “Frequency” formats)**


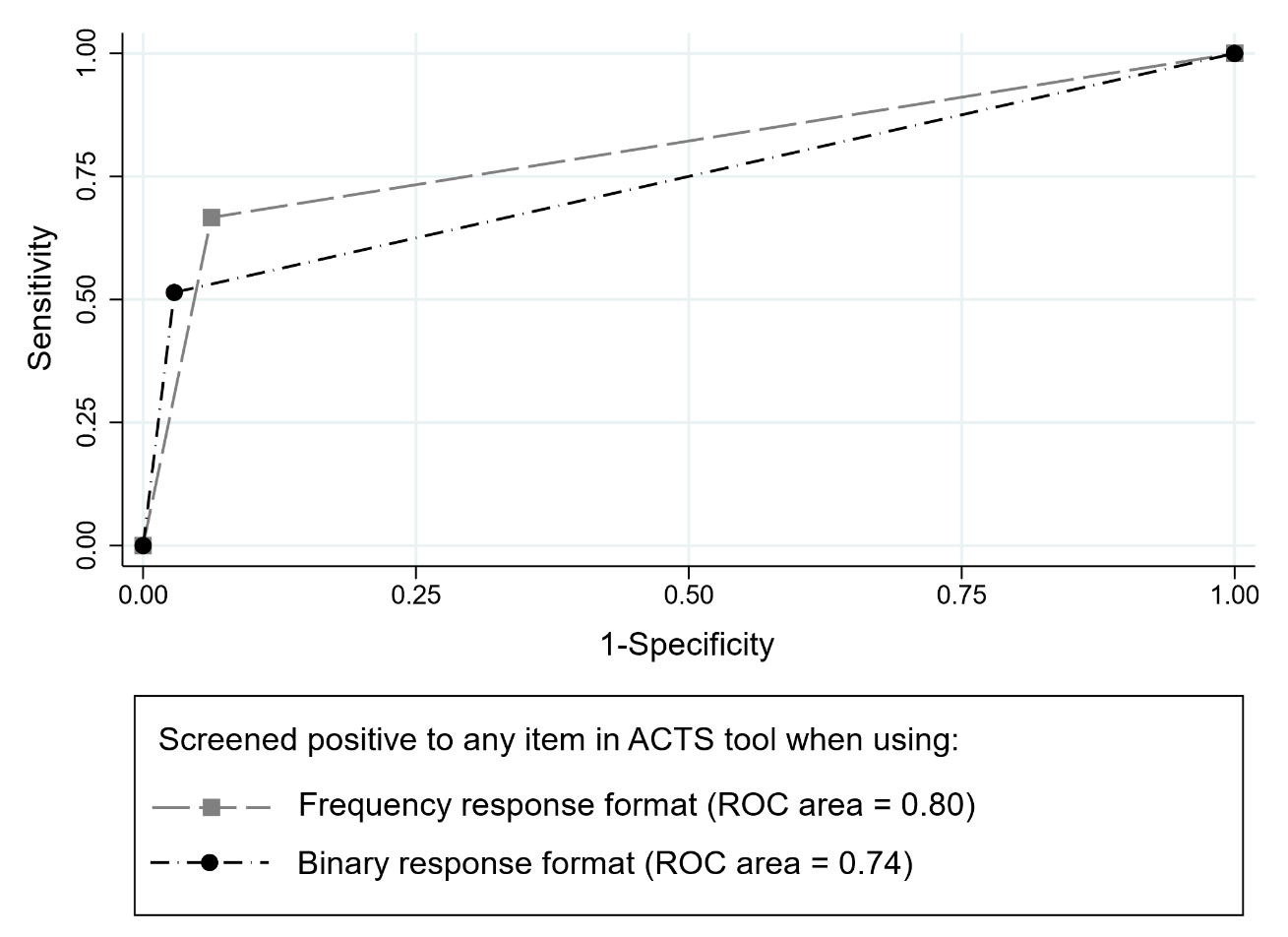

Supplement: Supplementary file 1 — Additional file 1: Figure 1. Receiver Operating Characteristic curves for the ACTS screening tool (binary “Yes/No” and Likert-scale “Frequency” formats). Description of data: Receiver Operating Characteristic curves for the ACTS screening tool when presented as binary “Yes/No” format and Likert-scale “Frequency” format [file 12889_2021_11781_MOESM1_ESM.docx]
